# Carotid-subclavian bypass or transposition extends the proximal landing zone for GORE thoracic branch endoprosthesis deployment

**DOI:** 10.1016/j.jvscit.2025.101960

**Published:** 2025-08-28

**Authors:** Jason Ejimogu, Daisy E. Martinez, Jeffrey Lu, Aakash Shah, Saurabh Gupta, Khanjan Nagarsheth, Shahab Toursavadkohi

**Affiliations:** Division of Vascular Surgery, Department of Surgery, University of Maryland, Baltimore, MD

**Keywords:** Thoracic aortic aneurysm, TBE, Endovascular, Debranching, TEVAR

## Abstract

Managing proximal aortic arch pathology remains complex. The thoracic branch endoprosthesis (TBE) offers a promising endovascular option. This retrospective case series evaluates TBE deployment after carotid-subclavian bypass and transposition in anatomically challenging patients. Ten patients underwent thoracic endovascular aortic repair with TBE between 2022 and 2025. Technical success was 100%, with no 30-day mortality, stroke, myocardial infarction, or respiratory complications. One patient required reintervention for a type III endoleak. The median patient age was 59 years, and the median hospital stay was 11.5 days. These findings support the safety and feasibility of TBE use with debranching in complex aortic arch repairs.

Thoracic aortic pathologies are life-threatening with high mortality.^1^ Thoracic endovascular aortic repair (TEVAR) is the current standard for thoracic abdominal aneurysms and type B dissections owing to lower morbidity and mortality rates than with open surgery.^1^ Management is more complex when disease extends into the aortic arch, particularly when involving supra-aortic branches. Hybrid approaches, combining cervical debranching with TEVAR, are often used in these cases.^2^ A common method is left carotid artery to left subclavian artery (LSCA) bypass or transposition, to allow safe coverage of the LSCA origin during graft deployment.^2^^,^^3^ For more proximal disease, open arch replacement remains the gold standard, but has higher stroke rates and mortality risk.^4^^,^^5^

The Gore Tag thoracic branch endoprosthesis (TBE) (W. L. Gore & Associates), approved by the US Food and Drug Administration in 2022, is a single-branch endograft for zone 2 and distal aortic pathology.^6^ However, off-label zone 1 use has been reported.^6^, ^7^, ^8^

Expanded cervical debranching strategies support broader TBE use in proximal arch pathology.^8^ Carotid-subclavian bypass and transposition reliably establish a proximal landing zone in patients not meeting anatomical criteria.^8^ This case series supports the feasibility and safety of carotid-subclavian bypass and transposition for TBE deployment in patients with complex arch anatomy. All patients provided consent for case publication.

## Methods

### Study design and data collection

A retrospective review was performed at a single tertiary center from 2022 to 2025. We included all patients undergoing carotid-subclavian bypass or transposition followed by TEVAR using a TBE graft in the arch. Selection criteria followed the Gore TBE instructions for use, such as a LSCA greater than 25 mm in length and adequate femoral access. One exception is that patients with a zone 2 length less than 20 mm were included if left common carotid artery-to-LSCA bypass or transposition achieved an adequate landing zone. Additionally, patients underwent simultaneous or staged repair based on anatomy. Aberrant anatomy, such as a right subclavian artery originating distal to the LSCA, required a staged procedure. All TBE branches were branched to the LSCA, with primary pathology in zones 2 or 3, but the proximal landing zone was in zones 1 and 2.

**Fig 1:**
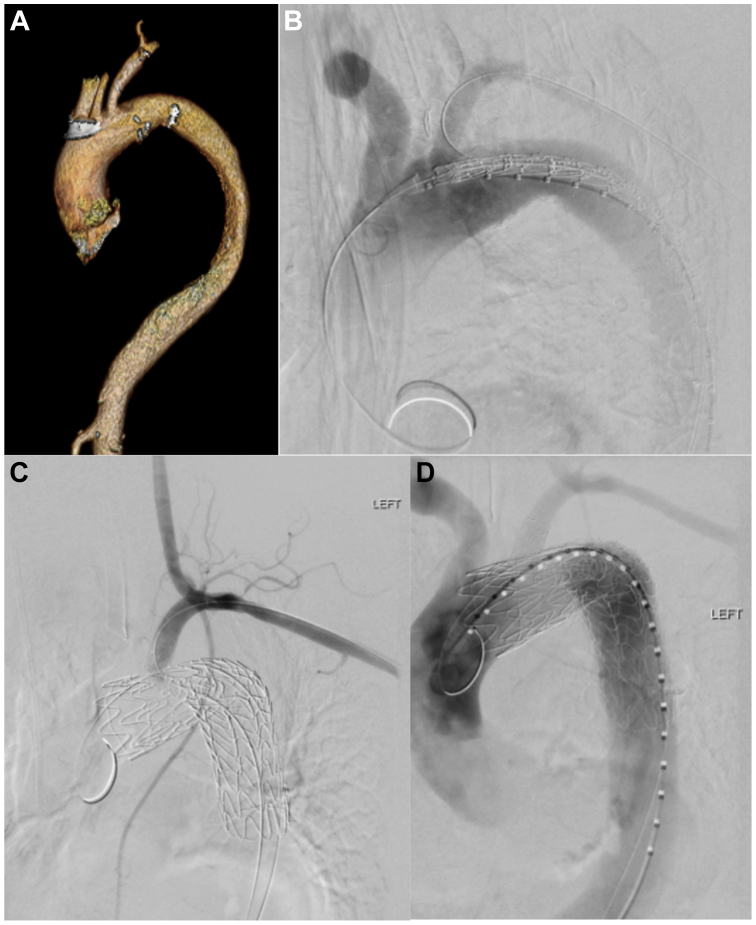
Left common carotid artery (LCCA) to left subclavian artery (LSCA) transposition with zone 1 thoracic branch endoprosthesis (TBE) deployment. **(A)** Preoperative three-dimensional reconstruction left anterior oblique image of the arch with head vessels demonstrating inadequate landing zone for standard zone 2 TBE. **(B)** Preimplant angiography left anterior oblique view of the TBE device loaded over both wires. **(C)** Angiography illustrating LCCA transposition patency. **(D)** Final angiography of aortic arch after implant of both TBE and LSCA branches.

For TBE deployment, vascular access was obtained percutaneously via left radial (6F sheath) and left common femoral (6F sheath, pigtail to ascending aorta) arteries. The 6F sheath was advanced from the left radial artery into the LSCA. The TBE device was deployed in zone 1 under fluoroscopic guidance, following confirmation with angiography (Fig 1). A previously snared wire accessed the TBE branch portal, and a covered stent was deployed into the LSCA. All grafts were oversized by 10% to 20% to ensure an adequate seal. No intraoperative transcranial Doppler examination was used. All patients had intraoperative angiography and motor evoked potential monitoring. Postoperatively, computed tomography angiography was performed in all patients.

**Fig 2:**
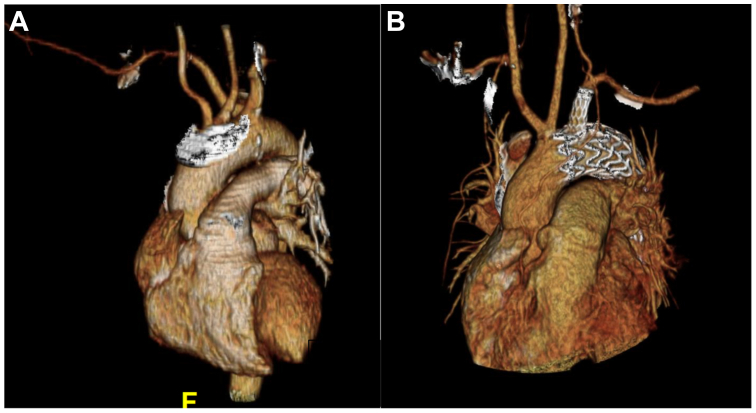
Aberrant right subclavian artery to right common carotid artery transposition with zone 1 thoracic branch endoprosthesis (TBE) deployment. **(A)** Preimplant three-dimensional (3D0 reconstruction of aortic arch and head vessels. **(B)** Postimplant 3D reconstruction with TBE and left subclavian artery (LSCA) branch.

For patients with aberrant anatomy undergoing a staged operation, a right subclavian-to-right common carotid transposition was done via a supraclavicular incision. The subclavian artery was divided proximal to the vertebral and anastomosed to the right carotid via side arteriotomy with 6-0 Prolene. The proximal right subclavian stump was ligated and there was no competitive flow. After cervical revascularization, a standard TBE deployment technique was used (Fig 2).

Data from electronic health records included demographics, comorbidities, length of stay, procedural indications, operative details (access site, bypass method, staging, time, and blood loss), discharge status, follow-up, and complications. The institutional review board approved the study and informed consent was obtained.

### Study outcomes

The primary outcome was technical success, defined as TBE graft placement with full pathology coverage. Secondary outcomes included absence of major adverse cardiovascular events, including cardiovascular death, nonfatal myocardial infarction, and stroke, within 30 days, and the absence of wound complications (infection, seroma, chyle leak, and nerve injury) and respiratory compromise requiring prolonged intubation.

## Results

A total of 10 patients underwent carotid-subclavian bypass or transposition with arch TBE placement. Demographic and comorbidity data are presented in Table I. The median age was 59 years (interquartile range [IQR], 52-73 years). The median length of stay was 11.5 days (IQR, 6-14 days), mainly affected by an inability to wean from ventilator, blood pressure control, and pain. Intraoperative details are provided in Table II. Procedure length included total operative time for staged repairs. The median procedure time was 239 minutes (IQR, 223-267 minutes). Six patients underwent simultaneous carotid-subclavian bypass or transposition, and four underwent staged procedures.

**Table I:** Demographic information, comorbidities, and indication for procedure

Patient	Age	Race	Sex	BMI	LOS	Past medical history and comorbidities	Indication for procedure
1	52	Black	Female	26.9	6	HTN	Aortic pseudoaneurysm
2	74	Black	Female	31.8	28	HTN, HLD, ruptured intracranial aneurysm 2011 s/p repair w/residual memory loss, ongoing nicotine use	Thoracic aneurysm
3	60	Black	Female	24.1	24	HTN, CAD, T2DM, HFpEF, AF, COPD	Complex type B dissection
4	58	Black	Female	33.6	11	HTN, HLD, CAD, T2DM, CHF	Symptomatic Kommerell diverticulum
5	73	Other	Male	30.5	6	HTN, HLD, CAD s/p CABG, HFrEF, CVA w/residual weakness, COPD, CKD	Aortic arch aneurysm
6	71	Black	Female	31.6	2	HTN, HLD, T2DM, CAD	Thoracic aneurysm
7	45	Black	Male	31.4	12	Prior aortic dissection s/p open repair w/axillo-aortic bypass, HTN, HLD, CAD, T2DM, CVA	Acute type A dissection
8	79	Asian	Male	24.3	13	HTN, CAD, T2DM	Thoracic aneurysm
9	57	Black	Female	43.3	4	HTN, HLD	Thoracic aneurysm
10	43	Black	Female	48.8	3	HTN, T2DM	Symptomatic Kommerell diverticulum

*AF,* Atrial fibrillation; *BMI,* body mass index; *CABG,* coronary artery bypass graft; *CAD,* coronary artery disease; *CKD,* chronic kidney disease; *CHF,* congestive heart failure; *COPD,* chronic obstructive pulmonary disease; *CVA,* cerebrovascular accident; *HFpEF,* heart failure with preserved ejection fraction; *HFrEF,* heart failure with reduced ejection fraction; *HLD,* hyperlipidemia; *HTN,* hypertension; *LOS,* length of stay; *T2DM,* type 2 diabetes mellitus.

**Table II:** Intraoperative details

Patient	Surgery type	Access site	Debranching	Staged repair?	Procedure length, minutes	Estimated blood loss, mL
1	Elective	Bifemoral	Aberrant RSCA – RCCA transposition	Yes	194	200
2	Elective	Bifemoral	LCCA – LSCA bypass w/prosthetic	No	426	700
3	Urgent	Bifemoral	LSCA – LCCA transposition	No	223	100
4	Elective	Bifemoral	Aberrant RSCA – RCCA transposition	Yes	184	150
5	Elective	Bifemoral	LCCA – LSCA bypass w/prosthetic	No	326	5
6	Elective	Bifemoral	Aberrant RSCA – RCCA transposition	Yes	237	50
7	Urgent	Bifemoral	LCCA – LSCA bypass w/prosthetic	No	267	300
8	Elective	Bifemoral	LCCA – LSCA bypass w/prosthetic	No	242	200
9	Elective	Bifemoral	LCCA – LSCA bypass w/prosthetic	No	241	300
10	Elective	Bifemoral	Aberrant RSCA – RCCA transposition	Yes	232	100

*LCCA,* Left common carotid artery; *LSCA,* left subclavian artery; *RCCA,* right common carotid artery; *RSCA,* right subclavian artery.

Technical success was achieved in all patients. There were no mortalities within or beyond 30 days. Additionally, there were no cases of major adverse cardiovascular events, surgical site wound complications, or respiratory compromise within 30 days. Further postoperative details and patient outcomes are summarized in Table III. In this cohort, only one patient experienced a complication requiring reintervention. Patient 5 had a type III endoleak on computed tomography angiogram postoperative day 2. The type III endoleak occurred between the main distal end of the TBE graft and the proximal TEVAR extension. The patient received a Gore Viabahn VBX (W. L. Gore & Associates) stent. A completion angiogram confirmed resolution of the endoleak. All patients were stable and discharged home.

**Table III:** Postoperative details and patient outcomes

Patient	Discharge status	Complications/MACE	Time from procedure to last follow-up, months	Vessel patent?	Need for reintervention
1	Alive	None	27	Yes	No
2	Alive	None	1	Yes	No
3	Alive	None	26	Yes	No
4	Alive	None	23	Yes	No
5	Alive	Type III endoleak	24	Yes	Yes
6	Alive	None	24	Yes	No
7	Alive	None	23	Yes	No
8	Alive	None	16	Yes	No
9	Alive	None	4	Yes	No
10	Alive	None	1	Yes	No

*MACE,* Major adverse cardiac event.

## Discussion

Aortic arch pathology is challenging owing to complex anatomy and high open repair risk. Conventional open arch repair, with hypothermic circulatory arrest and selective cerebral perfusion, remains the gold standard for suitable patients, but has a higher risk in comorbid patients with extensive disease and challenging anatomy. Current data on hybrid strategies report early mortality rates of 6% to 10%, comparable with open repair.^1^

Traditional TEVAR requires an adequate proximal landing zone. However, innovations in branched and fenestrated endografts expand eligibility. The Gore TBE preserves major branch perfusion with good outcomes.^5^ Liang et al^5^ reported 100% technical success and 97% freedom from reintervention at 3 years. Other arch-specific endografts are in development, but the Gore TBE is the most studied in the United States.^7^^,^^8^

Cervical revascularization, commonly carotid-subclavian or carotid-carotid bypass, is essential in hybrid and endovascular repairs. These procedures demonstrate greater than 90% patency at mid-term follow-up and low stroke rates.^2^^,^^3^ The timing of revascularization remains variable, with both staged and simultaneous approaches described. Retrospective data show no significant mortality or stroke differences between the two approaches, but further studies are needed.^9^

Careful patient selection is critical. In our series, patients were selected based on the Gore TBE instructions for use. Exclusion criteria were severely calcified or stenotic supra-aortic vessels and prior neck surgeries precluding safe cervical debranching. Key strategies to reduce risk are preoperative imaging to assess vessel patency, careful intraoperative cerebral monitoring, and routine completion angiography.

Combining carotid-subclavian bypass or transposition with TBE deployment allows for the treatment of complex arch disease. Favorable outcomes have been reported in experienced centers.^10^ One multicenter trial with 22 patients using the TBE in zone 2 had 100% technical success and no 30-day mortality, stroke, or paraplegia.^11^ As endovascular platforms continue to evolve, using fully endovascular, branched devices may be a valuable addition to a clinicians arsenal.

### Limitations

Despite being a relatively large series, we acknowledge that the small sample size limits the power and generalizability. The retrospective chart review nature limits accuracy as data depends on correct EHR input. However, our goal was to report our institutions early feasibility and outcomes, which will ultimately lead to larger comparative studies.

## Conclusions

Carotid-subclavian bypass and transposition are feasible and safe procedures involving the aortic arch for extending the proximal landing zone. This technique offers a reproducible approach that supports broader Gore TBE use in unfavorable arch anatomy.

## Funding

None.

## Disclosures

None.
